# Use of buccal mucosa grafts for urethral reconstruction in children: a retrospective cohort study

**DOI:** 10.1186/1471-2490-14-46

**Published:** 2014-06-05

**Authors:** Emilie K Johnson, Spencer I Kozinn, Kathryn L Johnson, Sohee Kim, David A Diamond, Alan B Retik

**Affiliations:** 1 Department of Urology, Boston Children’s Hospital, 300 Longwood Ave, Boston, MA 02115, USA

**Keywords:** Stricture, Hypospadias, Urethroplasty, Buccal mucosa, Oral mucosa

## Abstract

**Background:**

The use of buccal mucosa grafts (BMG) for urethral reconstruction has increased in popularity over the last several decades. Our aim was to describe our institutional experience with and outcomes after BMG urethroplasty.

**Methods:**

We conducted a retrospective cohort study of boys undergoing BMG urethral reconstruction. Preoperative and perioperative characteristics and postoperative outcomes were evaluated.

**Results:**

Twenty-nine patients (median age 8.2 years) underwent BMG urethroplasty from 1995–2012. Of the 10 patients undergoing 1-stage repairs, 6 had tubularized grafts, the last of which was performed in 2000 due to an unacceptably high revision rate (100%). A 2-stage approach was elected for 19 patients (median follow-up 21.3 months). Complications including stricture, fistula, or chordee were seen in 60% of patients completing both stages and 32% required ≥1 revision. However, 71% of 2-stage patients were free of significant problems at last follow-up.

**Conclusions:**

We found BMG to be a reasonable option for use in complex pediatric urethral reconstruction. Tubularized grafts had poor results, and we no longer use them. We favor a 2-stage approach for all patients except those with “simple” non-hypospadiac strictures. Although revision procedures were not uncommon, the majority of patients were ultimately free of long-term problems.

## Background

Urethral pathology in children is generally comprised of complex hypospadias cases and other causes of urethral stricture disease, which include traumatic, iatrogenic, and idiopathic etiologies. In many situations, these abnormalities can be reconstructed primarily. For hypospadias, most cases can be corrected via chordee release and local tissue rearrangement such as in the tubularized incised plate repair [1]. Short strictures less than 1–2 cm in length due to other causes are often amenable to direct visualization internal urethrotomy or primary anastomotic urethroplasty [2].

Hypospadias is most often repaired in the first 6–12 months of life with short-term success rates approaching 90% [3,4]. However, long-term complication rates may be much higher, particularly for patients with severe hypospadias where complication rates approaching 30% have been reported [5]. Complications include urethrocutaneous fistula, diverticulum, residual chordee, breakdown of the repair, and urethral stricture, all requiring further surgical intervention. Subsequent attempts at repair are less likely to succeed than primary intervention due to tissue loss and scarred, hypovascular, immobile skin. Additionally, patients with strictures longer than 1–2 cm and/or multiple strictures may require augmentation or replacement of the diseased urethral tissue to achieve a successful surgical result.

Over the last 2 decades, the use of replacement grafts has become an increasingly popular choice for cases with complex urethral pathology. In particular, buccal mucosa grafting (BMG) is an attractive option because the buccal surface is non-hair bearing, it normally exists in a moist environment, and the tissue is abundant and readily accessible. There is a robust body of literature regarding the use of oral mucosa in the adult urethral stricture population [6-8]. Both single and multi-staged approaches have been described with favorable outcomes thus far. However, less data exists for the use of BMG in the repair of complex urethral stricture disease pediatric patients. The aim of our study was to investigate and describe the Boston Children’s Hospital experience with BMG for urethral reconstruction.

## Methods

### Patient identification

We conducted a retrospective cohort study of all patients treated for urethral pathology at Boston Children’s Hospital whose repair required the use of BMG. Patients were initially identified via the presence of a Current Procedural Terminology (CPT) billing code for a complicated hypospadias repair (54340, 54344 or 54348), repair of hypospadias cripple (54352), first stage urethral revision (53400) and/or excision of oral mucosa for graft (40818). Patient operative notes were then screened to identify the use of a BMG during at least 1 urethral reconstruction procedure at our hospital.

### Data abstraction

Retrospective chart abstraction was performed for each patient in our cohort. Variables collected included demographics, urologic and non-urologic comorbidities, initial diagnoses, and pre-buccal repair characteristics (if applicable). We also collected perioperative data including type of repair, number of stages necessary, indication for the repair that included buccal mucosa, graft characteristics, immediate complications, and length of time between stages. We recorded duration of follow-up for each patient, need for (and types of) revision after buccal repair, assessed the proportion and types of long-term complications (e.g. stricture, fistula, chordee, etc.) and determined whether these problems persisted at the time of last visit to our clinic. Failure was identified by clinical history and examination, and (in some cases) flow rate. For 1-stage repairs, follow-up time was calculated as time since the BMG repair. For patients undergoing 2-stage repairs, follow-up time was calculated as the time since their 2^nd^ stage.

### Analysis

Descriptive statistics were used to characterize our population of patients undergoing BMG urethroplasty. Surgical characteristics and postoperative outcomes were evaluated for patients undergoing 1-stage and 2-stage repairs. Data analysis was conducted using IMB SPSS Statistics© Version 19, 2012, Somers, NY. The study was reviewed and approved by the Boston Children’s Hospital institutional review board. The protocol number is IRB-P00006955.

## Results

### Patient cohort

We identified 29 patients who underwent urethral reconstruction using buccal mucosa from 1995–2012 at Boston Children’s Hospital by a total of 11 surgeons. The demographic characteristics of these patients are illustrated in Table 1. For those who underwent an initial repair prior to their buccal urethroplasty, their median age at the time of first repair was 9.9 months. Seven patients had urologic comorbidities, including 1 with an unspecified 46 XY disorder of sexual differentiation and 4 patients with isolated cryptorchidism.

**Table 1:** Patient demographics (N = 29)

**Characteristic**	**N (%) or median [IQR]**
**Age at initial repair in months**	9.9 [6.8-18.6]
**Race**	
White	18 (62.1)
Black	4 (13.8)
Asian	1 (3.4)
Other or mixed	3 (10.3)
Unknown	3 (10.3)
**Urologic comorbidities (N = 7)**	
UDT	5 (71.4)
DSD	1 (14.3)
Vesicoureteral reflux	2 (28.6)
**Medical comorbidities (N = 14)**	
Pulmonary	4 (28.6)
GI	4 (28.6)
Cardiac	4 (28.6)
Endocrine	3 (21.4)
Other	2 (14.3)

Table 2 illustrates the characteristics of our patients prior to their buccal urethroplasty. The initial diagnosis was hypospadias in 24/29 patients (83%) and the initial meatal opening was proximal (proximal shaft, penoscrotal or perineal) in 18/24 cases (75%). Two patients with urethral strictures had a buccal urethroplasty as their first repair. Of the patients with prior repairs, 23/27 (81%) had at least one revision of their initial repair prior to BMG and 6/27 (22%) had 4 or more revisions.

**Table 2:** Pre-buccal graft repair characteristics

**Characteristic**	**N (%)**
**Initial diagnosis (N = 29)**	
Hypospadias	24 (82.8)
Urethral stricture	3 (10.3)
Urethral duplication	1 (3.4)
Chordee	1 (3.4)
**Initial hypospadias location (N = 24)**	
Distal	4 (16.7)
Midshaft	2 (8.3)
Proximal	18 (75.0)
**Initial type of repair (N = 27)**	
1 stage flap	4 (14.8)
1 stage tube	2 (7.4)
1 stage, unspecified type	1 (3.7)
First stage	1 (3.7)
Multistage	8 (29.6)
Unknown	5 (18.5)
Other	6 (22.2)
**Number of procedures prior to buccal grafting (N = 29)**	
0	2 (6.9)
1	5 (17.2)
2	5 (17.2)
3	5 (17.2)
4+	12 (41.4)
**Endoscopic management attempted prior to buccal grafting (N = 29)**	
Yes	12 (41.4)
No	17 (58.6)
**Buccal graft surgical approach**	
1-stage tube	6 (31.6)
1-stage onlay	4 (21.1)
2-stage	19 (65.5)

#### One-stage repairs

A 1-stage repair was elected for 10 patients in total. The clinical characteristics of these patients are illustrated in Table 3. For 6 patients, a tube graft was performed. All of these patients developed complications, with stricture being the most common, and all required at least 1 open revision. Due to these suboptimal outcomes, the last tube graft was performed at our institution in 2000. Since then, 4 patients have undergone 1-stage onlay BMG (3 ventral and 1 dorsal) with better results. Although ¾ have had a recurrent stricture, the only subsequent procedures required have been endoscopic stricture management in 2 patients.

**Table 3:** Clinical characteristics of patients undergoing 1-stage BMG urethroplasty (N = 10)

**Characteristic**	**N (%) or median [IQR]**
**Age at surgery in years**	9.6 [3.7-16.3]
**Duration of follow-up in months**	50.1 [10.6-120.2]
**Age at follow-up in years**	17.0 [11.3-19.7]
**Indication(s) for buccal repair**^**a**^	
Stenosis/Stricture	6 (60)
Breakdown	2 (20)
Diverticulum	1 (10)
Chordee	5 (50)
Fistula	1 (10)
**Graft length in cm**	3.5 [3.0-4.2]
**Surgical approach**	
Onlay	4 (40)
Tubularized graft – buccal only	5 (50)
Tubularized graft composite with bladder	1 (10)

^a^Numbers add to >100% due to patients with multiple indications for repair.

#### Two-stage repairs

A 2-stage approach was selected for 19 patients, whose clinical characteristics are illustrated in Table 4. Three patients are still awaiting their second stage procedures and 2 were recently completed with follow-up pending, so results are reported for the 14 patients who have undergone both stages and have subsequent follow-up. The median interval between stages was 9 months, and median follow-up was 21.3 months for patients undergoing both stages.

**Table 4:** Clinical characteristics of patients undergoing 2-stage BMG urethroplasty (N = 19)

**Characteristic**	**N (%) or median [IQR]**
**Age at 1**^**st **^**stage surgery**	8.1 [2.8-16.0]
**Duration of follow-up in months**^**a**^	8.2 [5.4-18.3]
**Age at follow-up in years**^**a**^	21.3 [6.9-37.7]
**Indication(s) for buccal repair**^**b**^	
Stenosis/Stricture	15 (79.0)
Breakdown	6 (31.6)
Diverticulum	5 (26.3)
Chordee	2 (10.5)
Fistula	6 (31.6)
**Graft length in cm**	3.8 [2.0-4.3]

^a^N = 14 patients who underwent both stages and had follow-up.

^b^Numbers add to >100% due to patients with multiple indications for repair.

Early complications (within 30 days of surgery) were seen in 3 patients after their 1^st^ stage and in 5 patients after their 2^nd^ stage. After the 1^st^ stage, we observed 1 patient with urinary retention, 1 with pyelonephritis, and 1 with a bolster (compressive dressing designed to protect graft) that became dislodged and required replacement under anesthesia. For the 2^nd^ stage, 2 patients had postoperative abscesses, and 1 had an early fistula.

When we defined a long-term complication as a problem such as stricture, fistula, diverticulum and/or chordee, 9/14 (60%) of patients undergoing a 2-stage repair had at least one of these over the course of their follow-up, with stricture being the most common. One patient undergoing a 2-stage repair required revision of the 1^st^ stage to correct glandular graft contracture at 9 months postoperatively, and 5/14 patients (36%) required at least 1 revision of their 2^nd^ stage repair, at a median of 13 months (range 10–37 months) after surgery. Median graft length was 3.0 cm in patients without vs. 3.5 cm in patients with a long-term complication. At the time of last visit to our clinic, 10/14 (71%) of patients were free of any complications such as stricture, fistula, or chordee.

### Surgical technique

Surgical technique was somewhat surgeon-specific; however, there were several common themes. The graft was secured to the recipient site with fine, absorbable suture, either running or interrupted based on surgeon preference. When possible, a vascularized interposition graft was used to cover the repair; 27% were dartos flaps and 73% required a tunica vaginalis flap for coverage. For 2-stage repairs the graft was secured in place with a bolster dressing, which was left in place for a median of 7 days. Urinary drainage was with a foley or urethral stent in 75%, a suprapubic catheter in 11%, and both a urethral and suprapubic catheter in the remainder (14%).

### Donor site

The BMG was harvested from the cheek in 27/29 (93%), 2 of whom had extension of the graft onto the lip. All grafts were harvested by the urologic surgeon. The remaining 2 patients had their graft harvested from the lip alone. All cheek donor sites were closed, and all lip donor sites were left open, a decision that was made by the surgeon to allow for potential re-harvest from the cheek if necessary in the future. No patient developed symptomatic donor site sequelae.

## Discussion

In this report, we detail our institutional experience with the use of BMG for urethral reconstruction, which spans nearly 3 decades. The majority of our patients had an initial diagnosis of hypospadias and required multiple reconstructions prior to presentation at our institution. Nearly 2/3 of patients underwent a 2-stage repair, and we favored this approach over time. Tube grafts were abandoned relatively early in our experience due to an unacceptably high re-stricture rate, which we hypothesize is due to circumferential graft contracture. Long-term complications were seen in 47% of patients undergoing 2-stage repairs and 32% required at least 1 revision of their BMG. However, 68% were free of significant problems at the time of last follow-up.

Buccal mucosa first gained popularity as an option for complex urethral reconstructions in the early 1990s [9,10]. BMG is an attractive option due to the fact that it is a mucosal, non-hair bearing surface with fast uptake and vascularization after free grafting for urethral substitution [10,11]. BM tissue is abundant and accessible and the donor site morbidity is acceptably low [12,13], although contractures have been reported with lip donor sites [14], as well as with large cheek grafts [15].

Multiple previous authors have reported their institutional experiences with buccal mucosa grafting for complex hypospadias. These reports have noted complication rates ranging from 14-57% [16-20], with most complications occurring early (within 6–12 months) after buccal repair [19,20]. Although our early experience with buccal grafts had a complication rate higher than the range previously noted in the literature, our more recent experience is congruent with that of other centers.

In addition to the learning curve inherent with a new technique, our suboptimal early results are reflective of the lack of success of tube grafting compared with other approaches. A consensus is forming in the literature that buccal tube grafts represent a less successful strategy than onlay BMG. Consistent with our experience, Hensle and colleagues’ report of a 50% complication rate with tube grafts [19], comparable to our rate of 100%. Surprisingly, Metro and colleagues noted opposite results in their series of 29 patients, where 42% of patients with onlay grafts developing postoperative strictures compared with only 6.3% of tube grafts [17].

Given our disappointing experience with tube grafts, we have adopted a philosophy of using onlay grafts for “simple” strictures, and have come to favor a 2-stage repair for the majority of patients requiring a revision urethroplasty. Newer approaches to onlay grafting, such as the widely anchored patch recently suggested by Djordjevic and colleagues may improve the results of a 1-stage approach for these complicated patients [21]. Thus far, we have found buccal mucosal grafting to be unnecessary for primary hypospadias repairs, although other authors have reported success with this technique for cases of hypospadias with severe chordee [22].

Alternatives to BMG do exist for patients with complex urethral stricture disease where local tissue is not available or suitable for reconstruction. For example, one alternative is to use a posterior auricular full-thickness skin graft as a material for urethral substitution. Our group has recently explored this concept for patients who require reconstruction of the distal penile urethra where buccal mucosa from the cheek might be thicker than desired. Additionally, the use of posterior auricular grafting has been reported in adults where the oral mucosa was unsuitable due to fibrotic changes [23].

Our study has several limitations that warrant mention. Although our experience spans a significant timeframe, it represents a single-institution, retrospective description that may have limited generalizability outside of the complex group of patients treated at our tertiary care academic pediatric hospital. Additionally, this series represents the experience of 11 different surgeons (average cases 2.6/surgeon), underscoring the fact that complex urethral pathology is relatively rare and difficult to manage even in a tertiary care setting. It is also difficult to make specific conclusions regarding the role of a specific surgeon on outcomes due to the small number of patients per surgeon. Also, our patient cohort was identified using billing data, so we could have unintentionally omitted patients undergoing buccal grafting at our institution. To mitigate this limitation, we were purposely broad in the initial billing codes included, and then manually screened patients to exclude them from the final study population. Due to the retrospective nature of our study, we were also limited in the outcome data that could be collected from the patient record; the same outcome measures were not necessarily reported in all encounters for each patient, and we were unable to systematically assess patient-reported outcomes such as quality of life (QOL) and sexual function, which have only been comprehensively reported by one previous group [14,24]. Although these studies suggest that QOL and sexual function was favorable among patients at a single center, the incorporation of patient-reported outcome data from additional centers into future reports is paramount. In particular, assessment of post-pubertal outcomes will be of great interest. Finally, our series represents a heterogeneous group of patients, surgeons, and approaches. While this allowed a basic evaluation of several surgical approaches, this heterogeneity of our patients made a detailed determination of specific patient-and surgeon-level factors predicting complications after surgery challenging.

Despite these limitations, our study provides additional insight into the experience using BMG for reconstruction in patients with complex urethral pathology treated at a specialized academic center. Specifically, our series adds to the knowledge regarding complication rates and particular difficulties with tubularized 1-stage repairs. Our data should help to facilitate appropriate expectation setting for patients and families regarding complications and the potential for revision after buccal urethroplasty, and highlights the importance of long-term follow-up.

## Conclusions

At our institution, we have found BMG to be a reasonable option for use in complex urethral reconstruction in children where local tissue is not available or suitable. Tubularized grafts had poor results in our series, and we no longer use them. Currently we favor a 2-stage approach for all patients except for those with “simple” urethral strictures due to causes other than hypospadias. Although many patients required revision procedures after BMG, the majority were free of long-term problems at the time of last follow-up.

## Consent

This study was reviewed and approved by our hospital’s institutional review board. The results are reported in aggregate, and no individual case details were reported. Therefore a waiver of informed consent was granted for this study.

## Abbreviations

BMG: Buccal mucosa graft; QOL: Quality of life.

## Competing interests

The authors declare that they have no competing financial or non-financial interests.

## Authors’ contributions

EKJ contributed to the study design, performed data collection, planned and executed the analysis, drafted and revised the manuscript and approved the final version. SIK contributed to the study design, performed data collection, and assisted with drafting and revising the manuscript and approved the final version. KLJ performed data collection, preliminary data analyses, assisted with drafting and revising the manuscript and approved the final version. SK contributed to the study design, performed data collection, and assisted with drafting and revising the manuscript and approved the final version. DAD supervised the study design and interpretation of results, assisted with drafting and revising the manuscript and approved the final version. ABR supervised the study design, data collection and interpretation of results, assisted with drafting and revising the manuscript and approved the final version.

## Pre-publication history

The pre-publication history for this paper can be accessed here:

http://www.biomedcentral.com/1471-2490/14/46/prepub
